# ICG fluorescence imaging as a new tool for optimization of pathological evaluation in breast cancer tumors after neoadjuvant chemotherapy

**DOI:** 10.1371/journal.pone.0197857

**Published:** 2018-05-25

**Authors:** Isabelle Veys, Catalin-Florin Pop, Romain Barbieux, Michel Moreau, Danielle Noterman, Filip De Neubourg, Jean-Marie Nogaret, Gabriel Liberale, Denis Larsimont, Pierre Bourgeois

**Affiliations:** 1 Service of Surgery, Institut Jules Bordet, Université Libre de Bruxelles, Brussels, Belgium; 2 Service of Nuclear Medicine, Institut Jules Bordet, Université Libre de Bruxelles, Brussels, Belgium; 3 Statistics Department, Institut Jules Bordet, Université Libre de Bruxelles, Brussels, Belgium; 4 Department of Pathology, Institut Jules Bordet, Université Libre de Bruxelles, Brussels, Belgium; University of Campinas, BRAZIL

## Abstract

**Background:**

Response to neoadjuvant chemotherapy (NACT), particularly pathologic complete response (pCR), is an independent predictor of favorable clinical outcome in breast cancer (BC). The accuracy of residual disease measurement and reporting is of critical importance in treatment planning and prognosis for these patients. Currently, gross pathological evaluation of the residual tumor bed is the greatest determinant for accurate reporting of NACT response. Fluorescence imaging (FI) is a new technology that is being evaluated for use in the detection of tumors in different oncological conditions.

**Objective:**

The aim of this study was to evaluate whether indocyanine green fluorescence imaging (ICG-FI) is able to detect residual breast tumor tissue after NACT in breast surgical operative specimens.

**Methods:**

Patients who underwent NACT for BC and were admitted for breast surgery were selected for participation in this study. Free ICG (0.25 mg/kg) was injected intraoperatively. Tumor-to-background fluorescence ratio (TBFR) was calculated on ex vivo samples from the surgical specimen.

**Results:**

One hundred and seventy-two samples from nine breast surgical specimens were evaluated for their fluorescence intensity. Among them, 52 were malignant (30.2%) and 120 were benign (69.8%). The mean TBFR was 3.3 (SD 1.68) in malignant samples and 1.9 (SD 0.97) in benign samples (p = 0.0002). With a TBFR cut-off value of 1.3, the sensitivity, specificity, negative predictive value, false negative rate, and false positive rate of ICG-FI to predict residual tumoral disease in breast surgical samples post-NACT were 94.2%, 31.7%, 92.7%, 5.8%, and 68.3%, respectively. If we restricted our analysis to only patients who achieved pCR, the negative predictive value for ICG-FI was 100%.

**Conclusions:**

These first observations indicate that ex vivo ICG-FI is sensitive but not sufficiently specific to discriminate between benign breast tissue and malignant residual tissue. Nevertheless, its negative predictive value seems sufficiently accurate to exclude the presence of residual breast tumor tissue on the operative specimen of patients treated by NACT, representing a potential tool to assist pathologists in the assessment of breast surgical specimens.

## Introduction

Breast cancer (BC) is by far the most frequently diagnosed malignant cancer among women worldwide [1]. Despite the fact that early BC detection strategies are in place in Western countries, almost a third of all BCs are diagnosed with advanced stage disease at presentation, requiring neoadjuvant treatment [1,2], and at least 7% of women with localized disease will also benefit from neoadjuvant treatment [3].

Historically, the primary goals of neoadjuvant chemotherapy (NACT) have been to improve surgical options by downsizing the tumor before surgery, to provide a therapeutic alternative for patients with unresectable disease, and to reduce the extent of surgery needed to achieve adequate resection [2,4]. NACT also offers several other advantages. For example, it is a unique opportunity for the evaluation of treatment response and individualized therapy, providing prognostic information that allows clinicians to change or discontinue treatment in the case of unresponsive tumors. This evaluation is also useful in a research setting where response to NACT is utilized as a surrogate endpoint in many clinical trials [3]. In addition, it offers the possibility to collect tumor samples before, during, and after treatment, facilitating translational research to identify markers of response [4].

NACT trials have revealed the phenomenon of pathologic complete response (pCR) that has been reported to be an independent predictor of favorable clinical outcomes for all molecular subtypes of BC [3]. The overall rate of pCR attainment is relatively low (22%) and, consequently, the goal of increasing the rate of pCR has become the end point of neoadjuvant trials with an expectation that this will improve overall survival [2–4].

Response to NACT can be assessed by clinical examination, breast imaging techniques, and, mainly, by histopathologic examination of the surgical specimen that remains the gold standard for evaluation and for reporting tumor response to NACT. Grading of tumoral response is of critical importance in treatment planning and prognosis of these patients. Currently, the accuracy of detection of residual disease at pathology depends on the correct localization and sampling of the suspected “residual tumor bed” on the operative specimen by gross pathologic methods that represent the single greatest determinant for accurate reporting of pCR [2–5].

Fluorescence imaging (FI) using indocyanine green (ICG), a nonspecific fluorophore, has been reported to be a new promising noninvasive technology for the detection of various primary tumors including hepatocarcinoma, head and neck cancers, lung cancers, and brain tumors [6–9]. Hence, ICG-FI has been evaluated as a new imaging technique for the detection of various metastatic deposits such as lymph nodes, and hepatic and peritoneal metastases [10–14]. The mechanism for preferential uptake of ICG in tumor tissues is not fully understood. The most likely hypothesis involves the enhanced permeability and retention (EPR) effect observed in tumoral tissue secondary to neoangiogenesis [13,15]. If injected intravenously, ICG molecules bind to serum proteins in vivo. In tumoral tissue, protein-bound ICG molecules accumulated in the extravascular space can emit fluorescence (peak at 840 nm) under near-infrared illumination, and the fluorescence signals emitted can be visualized through connective tissue 5–10 mm thick. As the half-life of ICG in blood circulation is 3–5 minutes, ICG is rapidly washed-out from the intravascular space and the extravascular accumulation of ICG will be responsible for the observed hyperfluorescence of tumoral tissue in contrast to surrounding normal tissue [12,13,16].

Ntziachristos and colleagues were the first to report the detection of BC by ICG-FI after ICG intravenous (IV) injection [17]. The use of in vivo ICG-FI has been reported for surgical margin evaluation and primary tumor identification in BC but these data are very limited and addressed only cases of early BC [18,19]. There are no data about the role of ICG-FI for the detection of residual BC tissue after NACT.

On these bases, we hypothesized that ex vivo ICG-FI of breast surgical specimens after intraoperative ICG IV injection could improve the detection of residual tumoral tissue in patients treated for BC after neoadjuvant chemotherapy and help to guide the pathologist in operative specimen sampling for further standard pathological examination.

## Patients and methods

### Study and patients

This was an exploratory study approved by the Investigational Review Board of the Institut Jules Bordet (CE 2179) and registered with the Clinical Trials.gov Protocol Registration System (NCT02032563**:** https://register.clinicaltrials.gov) (EUDRACT 2013-004496-12). All patients provided written informed consent before inclusion in the study. The primary objective of the study was to evaluate whether the ex vivo ICG-FI technique is feasible and able to detect residual BC tissue on operative specimens (paraffin embedding breast samples) according to the fluorescence intensity of tumoral lesions, scars, and healthy tissue in patients treated with surgery after NACT. Exclusion criteria were those mentioned in our previous ICG-FI studies [10–12, 14].

### Preoperative and operative treatment

All patients received NACT after a multidisciplinary oncologic consultation, according to standard protocols based on national and international guidelines (anthracycline- and taxane-based regimens; HER2-positive tumors were treated with a trastuzumab-based regimen). All patients underwent mastectomy due to their locally advanced disease.

### Histopathologic evaluation and assessment of residual disease

Complete macroscopic and microscopic examination, and immunohistochemistry were performed using a routine clinical procedure according to international guidelines. Pathologic examination was performed by an experienced breast pathologist (DL). Two systems of post-neoadjuvant pathologic tumor staging—Residual Cancer Burden (RCB) and the American Joint Committee on Cancer post-neoadjuvant therapy staging system (ypTNM) were used [20,21]. We defined pCR as histopathologic complete absence of invasive tumor cells in all breast specimens removed as part of mastectomy (ypT0).

### Fluorescence imaging

**Fig 1:**
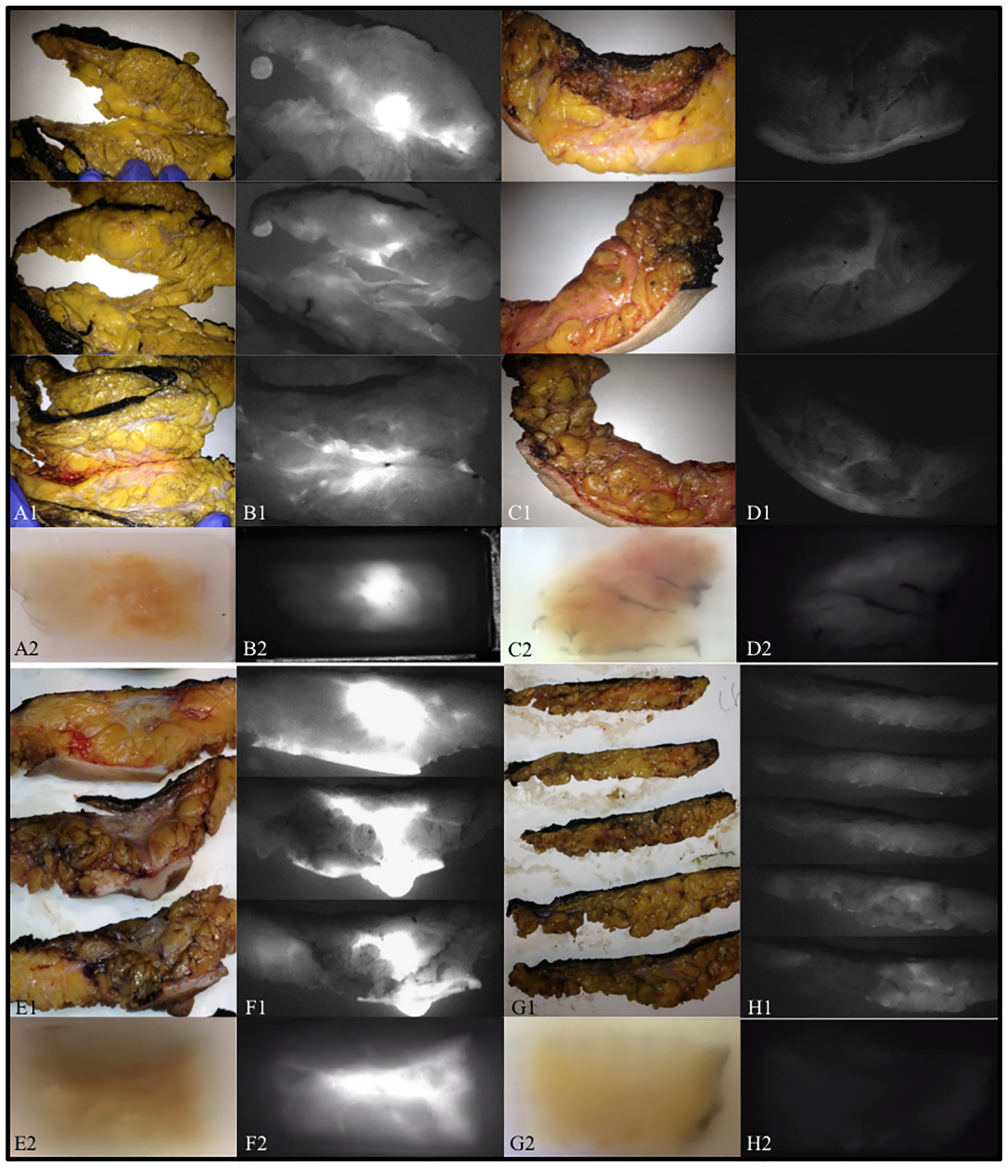
Breast surgical specimens during gross pathology preparation in standard white light (A1, C1, E1, G1) and by fluorescence imaging, for patient with residual disease (P5 and P4) and hyperfluorescence (B1 and F1) and for patient with a pCR (P6 and P3) and hypofluorescence (D1 and H1). Corresponding breast samples in paraffin-embedded blocks of selected patients (P5, P3, P4, P6) under white light (A2, C2, E2 and G2). The same samples under fluorescence imaging showing hyperfluorescence (B2 and F2) and hypofluorescence (D2 and H2).

Free ICG (0.25 mg/kg; Pulsion Medical System, Belgium) was injected through a peripheral venous line at induction time of anesthesia. Surgical breast operative specimens were imaged during macroscopic pathological evaluation of the specimen in the pathology department (see Fig 1A1–1H1) and further FI was performed on blocks (breast samples) after paraffin embedding (PE) (see Fig 1A2–1H2).

Ex vivo ICG-FI was performed using a dedicated near-infrared NIR camera system, Fluobeam 800 (Fluoptics, Grenoble, France) an integrated NIR light source with a maximum absorption between 750 nm and 800 nm and a maximum emission between 780 nm and 850 nm. The excitation was provided by a class 1 expanded laser source at 780 nm. The fluorescence signal was collected by a CCD through a high pass filter with high transmittance for wavelength >830 nm. The working distance of video recorded was fixed at 15 cm and the acquisition time of fluorescent signal was set to 167 milliseconds. All specimens were imaged in standard conditions with the camera by two investigators (RB, FCP).

The fluorescence intensity of each PE sample from mastectomy operative specimens was evaluated according to two methods using FI videos of the paraffin blocks. In the first method, a visual evaluation (visual scale) was done by one investigator (FCP) and the samples were classified according to their fluorescence as not-fluorescent (defined as samples with little or no fluorescence compared with the surrounding tissue), or as fluorescent (defined as samples with obvious fluorescence compared with the surrounding tissue). In the second method, a semi-quantitative analysis of the fluorescence intensity of each sample was performed using tumor-to-background fluorescence ratio (TBFR) calculations (quantitative scale). For each specimen, regions of interest (ROIs) were delineated over the suspected tumor sample (numerator) and on the adjacent normal breast tissue (denominator). For each ROI, fluorescence intensity, expressed in Arbitrary Units (AUs), was measured with the IC-Calc 2.0 program.

### Residual tumor assessment by ICG-FI

To further quantify residual tumor assessment prediction by ICG-FI, the following parameters were assessed: sensitivity (Se), specificity (Sp), negative predictive value (NPV), false negative rate (FNR), and false positive rate (FPR). To analyze the complementary contribution of ICG-FI for residual tumor assessment, histopathological slides of fluorescent samples were reviewed by an experimented pathologist (DL). Furthermore, for the samples that were classified as hyperfluorescent but with normal benign features at first histopathological examination, serial sections at 150, 300, and 450 μm were performed on paraffin blocks to exclude the presence of residual malignant fluorescent tissue within the sample.

### Statistical analyses

Fluorescence intensity was assessed using visual (clinical) and quantitative scales. TBFR of the mastectomy samples was analyzed as a continuous and categorical variable using a cut-off of 1.3 (<1.3 / ≥ 1.3). The cut-off threshold value of 1.3 was determined to provide the best agreement between the visual and the quantitative scale. As the size of the sample was not the variable of interest but only an adjustment variable, the analysis was performed in a continuous way. Because the unit of analysis was the mastectomy sample and not the patient, and the data are not independent, we used the Generalized Estimating Equations (GEE) model (for continuous and categorical variables) in order to take into account the correlation structure within the patient. In the GEE analyses, empirical instead of model-based standard errors were used because they are more robust against misspecification of the correlation structure. An exchangeable covariance matrix was used. Fluorescence was analyzed in univariate GEE.

## Results

### Patients and tumor characteristics

Between July 2013 to October 2014, eight women treated with NACT for BC were enrolled to participate in this study. One patient had bilateral BC. The mean age was 52.4 years (range, 31–65 years). There were 8 invasive ductal carcinomas (IDC) and 1 invasive lobular carcinoma (ILC). According to intrinsic subtype classification, 7 BCs were luminal B tumors and 2 were triple-negative (TN) tumors. Seven patients had locally advanced BC, with clinical T3 (4 patients) or T4 (3 patients) disease, and 2 of them had metastatic disease. Four BCs were multifocal. For 7 patients, the NACT was an anthracycline- and taxane-based regimen, one patient had 6 courses of 5-fluoro-uracil (5-FU) in association with cisplatin regimens. There were 4 patients with human epidermal growth factor receptor 2 (HER2) positive tumors, 3 were treated with a trastuzumab-based regimen, and 1 with a trastuzumab emtansine regimen. A complete imaging response using magnetic resonance imaging (MRI) was reported in 3 patients and final histopathological examination confirmed a pCR in 4 patients. Clinical and tumoral characteristics of the evaluated patients are detailed in Table 1.

**Table 1:** Patient and tumor characteristics: Breast tumor characteristics of study patients and their response to neoadjuvant chemotherapy evaluated by MRI and final pathology.

Pat. ID°	Histology	Tumor Grade	Intrinsic Subtype	MRI Response to Neoadjuvant Chemotherapy	Residual Tumor Bed in mm	ypTNM	RCB	Samples (Samples +)
**1**	**IDC**	**3**	**TN**	**PR**	**60**	**ypT3N0**	**2**	**17 (11)**
**2**	**ILC**	**2**	**Lum B HER2-**	**SD**	**130**	**ypT3(m)N3a**	**3**	**19 (17)**
**3**	**IDC**	**3**	**LumB HER2-**	**CR**	**90**	**ypT0N0**	**pCR**	**30 (0)**
**4**	**IDC**	**3**	**LumB HER2+**	**ND**	**52**	**ypT3N1a**	**3**	**15 (5)**
**5**	**IDC**	**2**	**LumB HER2+**	**PR**	**40**	**ypT2(m)N3a**	**3**	**16 (15)**
**6**	**IDC**	**3**	**TN**	**PR**	**100**	**ypT0is(m)N0**	**pCR**	**13(1**^**is**^**)**
**7**	**IDC**	**3**	**LumB HER2+**	**CR**	**80**	**ypT1bN0**	**1**	**19 (1)**
**8**^**R**^	**IDC**	**3**	**LumB HER2+**	**PR**	**55**	**ypT0is(m)N0**	**pCR**	**19(1**^**is**^**)**
**8**^**L**^	**IDC**	**3**	**LumB HER2-**	**CR**	**70**	**ypT0isN0**	**pCR**	**24(1**^**is**^**)**

Pat.ID, patient identifier; R, right breast; L, left breast; IDC, invasive ductal carcinoma; ILC, invasive lobular carcinoma; TN, triple-negative; Lum B, Luminal B; HER2, human epidermal growth factor receptor 2; MRI, magnetic resonance imaging; CR, complete response; PR, partial response; SD, stable disease; ND, not done; ypTNM, pathological classification post neoadjuvant treatment of tumor—nodes—metastasis (see ref. [21]); m, multifocal; is, ductal carcinoma in situ; RCB, residual cancer burden; pCR, pathological complete response;

^+^, malignant samples;

### ICG-FI and visual scale analysis

Across the 8 patients, a total of 196 PE samples (blocks) from 9 mastectomy operative specimens were analyzed by histopathology. One hundred and seventy-two of them were evaluated for fluorescence intensity (87.8%). Twenty-four samples could not be evaluated for their fluorescence intensity due to the proximity of high fluorescent background from nipple (n = 9) or breast skin (n = 15). The median time between ICG injection and mastectomy specimen resection was 90 minutes (mean: 89.2 min, range: 47–135 min). There was residual tumoral tissue in 52 samples (30.2%) and no residual tumoral tissue in 120 (69.8%) at final histopathology. The sizes of malignant and benign mastectomy samples were not significantly different, 24.4 mm (SD 4.7) and 22.5 mm (SD 5.6) (p = 0.89), respectively. Mastectomy sample characteristics are detailed in Table 2. According to visual evaluation, 56 samples (32.4%) were classified as nonfluorescent. The sensitivity and specificity were 94.2% (49/52) and 44.2% (53/120), respectively. The NPV, FNR, and FPR of ICG-FI in predicting residual tumoral cells in mastectomy specimens were 94.6% (53/56), 5.8% (3/52), and 55.8% (67/120), respectively.

**Table 2:** Pathological and fluorescence imaging characteristics of mastectomy samples: Characteristics of mastectomy samples evaluated by indocyanine green-fluorescence imaging after standard pathological evaluation of the 9 breast tumor surgical specimens.

Mastectomy Samples	Total	Malignant n %		Benign n %		p
**Number**	**172**	**52**	**30.2%**	**120**	**69.8%**	**NA**
**Size (in mm)**						
**Mean (SD)**	**23.1(5.4)**	**24.4 (4.7)**		**22.5 (5.6)**		**0.89**
**Type of Tumor**						**< 0.0001**
**IDC**	**153**	**35**	**22.9**	**118**	**77.1**	
**ILC**	**19**	**17**	**89.5**	**2**	**10.5**	
**Grade of Tumor**						
**2**	**35**	**32**	**91.4**	**3**	**8.6**	**< 0.0001**
**3**	**137**	**20**	**14.6**	**117**	**85.4**	
**Intrinsic Subtype**						
**Luminal B HER2-**	**73**	**18**	**24.7**	**55**	**75.3**	**0.99**
**Luminal B HER2+**	**69**	**22**	**31.9**	**47**	**68.1**	
**Triple Negative**	**30**	**12**	**40**	**18**	**60**	
**Microscopic Scaring**						
**Yes**	**45**	**17**	**32.7**	**28**	**23.3**	**0.39**
**No**	**127**	**35**	**67.3**	**92**	**76.7**	
**Samples from Patient with**						
**pCR**	**86**	3*	**3.5**	**83**	**96.5**	**NA**
**No-pCR**	**86**	**49**	**57**	**37**	**43**	
**Visual Fluorescence Scale**						**< 0.0001**
**Hyperfluorescent**	**116**	**49**	**42.2**	**67**	**57.8**	
**Nonfluorescent**	**56**	**3**	**5.4**	**53**	**94.6**	
**Fluorescence in AUs**						**< 0.0001**
**Mean (SD)**	**9.1(9.6)**	**15.8(13.87)**		**6.2(4.83)**		
**TBFR**						**< 0.0001**
**Mean (SD)**	**2.3(1.4)**	**3.3(1.68)**		**1.9(0.97)**		

p, p value; SD, standard deviation; NA, not applicable; IDC, invasive ductal carcinoma; ILC, invasive lobular carcinoma; HER2, human epidermal growth factor receptor 2; pCR, pathological complete response;

*, ductal carcinoma in situ only; AUs, arbitrary units; TBFR, tumor-to-background fluorescence ratio.

### Quantitative scale analysis of ICG-FI in mastectomy samples

Among the 172 analyzed mastectomy samples imaged by ICG-FI, the mean of maximal fluorescence was 15.8 AUs (SD 13.9) in pathologically positive samples and 6.2 AUs (SD 4.8) in negative samples (p <0.0001). The mean TBFR was 3.3 (SD 1.68) in malignant samples and 1.9 (SD 0.97) in benign samples (p = 0.0002). With a cut-off TBFR value of 1.3, the Se, Sp, NPV, FNR, and FPR of ICG-FI in predicting residual tumoral disease in mastectomy samples post-NACT were 94.2% (49/52), 31.7% (38/120), 92.7% (38/41), 5.8% (3/52), and 68.3% (82/120), respectively.

The three false-negative ICG-FI samples (B10, B19.2, and B20) were found in patient identifier (ID) 1 with an IDC subtype TN with high Ki 67 value (at 95%) and high malignant cellularity (95%, 100%, and 85%). Table 3 shows the crosstabs between fluorescence and mastectomy sample status among the entire sample population. If we restricted our analysis to the cases with a histopathological pCR, the NPV, FNR, and FPR in predicting pCR in mastectomy samples post-NACT were 100% (29/29), 0% (0/3), and 65.1% (54/83), respectively.

**Table 3:** Fluorescence intensity (Visual and quantitative scale) by mastectomy sample status and ICG-FI accuracy.

		Mastectomy Sample Status			ICG-FI Accuracy		
Fluorescence		Malignant	Benign	Total	Se	Sp	NPV
**Visual Scale**	**Positive (fluorescent)**	**49**	**67**	**116**	**94.2**	**44.2**	**94.6**
	**Negative (nonfluorescent)**	**3**	**53**	**56**			
**TBFR**	**Positive (ratio ≥ 1.3)**	**49**	**82**	**131**	**94.2**	**31.7**	**92.7**
	**Negative (ratio < 1.3)**	**3**	**38**	**41**			

TBFR, tumor-to-background fluorescence ratio; ICG-FI, indocyanine green-fluorescence imaging; Se, sensitivity; Sp, specificity; NPV, negative predictive value.

On univariate analyses (for malignancy), intrinsic subtype, the presence of scar cells at pathology, and mastectomy sample size were not significantly different between malignant and benign samples. Conversely, the type and grade of tumor, as well as the fluorescence intensity evaluation performed by visual scale and by quantitative scale, were statistically different between malignant and benign samples (Table 2). After adjustment for size, type, and grade, fluorescence evaluation remained statistically associated with malignancy of the lesion.

### ICG-FI and secondary histopathological evaluation for residual tumor assessment

The second histopathological evaluation of mastectomy samples guided by ICG-FI failed to detect any additional residual malignancy. Additional analyses by serial section performed on PE blocks that presented with hyperfluorescence and benign normal features (without benign breast lesions) were done on 17 samples from 6 mastectomy specimens. The histopathological examination was performed by the same pathologist (DL). Serial sectioning found, in one case, an additional residual invasive tumor in patient ID 7, sample A02, but this was not related to the fluorescence of the sample. The tumor cellularity of this additional malignant focus was only 1%. These additional findings did not change the RCB 1 status of the initial histopathological report.

## Discussion

Neoadjuvant chemotherapy has not yet demonstrated a definitive benefit over adjuvant therapy with regard to improving survival outcomes except in the patient subgroup with a pathological complete response to therapy at the time of surgical resection [22]. Precise assessment of breast tumor response after NACT is very important for these patients in whom a pCR can be accurately detected and remains the ultimate goal [4]. Clinical evaluation of NACT response is difficult, especially in tumors that have responded to therapy where it is difficult to assess the actual tumor size versus treatment-induced changes [23]. Current imaging techniques such as mammography and ultrasound are not considered adequate for assessment of tumor response. The response to NACT relies essentially on the use of MRI to monitor the disease [24] but histopathological examination of tumor response to NACT remains the gold standard for final evaluation and for reporting complete responses [2–5].

The cornerstone of good pathological assessment of breast surgical specimens after NACT is the identification of the area that correlates best with clinical and radiological findings. Radiological tumor marking (clips, carbon) before NACT helps, but cannot define the tumor bed accurately. Therefore, handling and gross (macroscopic) examination of the operative specimen remains the gold standard technique for the selection of suspected areas on the operative breast specimen for further standard pathological microscopic analyses. After slicing surgical specimens into 3–5 mm sections, the largest pretreatment area should be selected for sampling. The extent of tissue sampling varies from in “toto” (for small specimens < 5 cm) to 1 or 2 tissue blocks from every 1 cm of pretreatment tumor size (for specimens larger than 5 cm) [5], or 10 blocks at least from an entire specimen [25]. Because histologic patterns of residual breast tumor tissue after NACT are diverse, different sampling methods can yield different evaluation results, potentially resulting in sampling errors and inappropriate final histopathological reports [26].

Therefore, a standard approach for evaluating surgical specimens post-NACT is essential for an accurate evaluation of the pathological response that could be used as an indicator of treatment response to current and novel therapies. Since 2015, the recommendations specifically designed for standardized pathological evaluation and reporting of neoadjuvant BC operative specimens have advised that improvements in our ability to compare pathology results could provide better personalized cancer therapy [5, 26].

Many imaging techniques have been used for evaluation of tumor changes after NACT, mainly functional imaging techniques such as dynamic contrast-enhanced MRI, diffusion-weighted MRI, and nuclear imaging (with 18-FDG) [24, 27, 28]. MRI is recognized as the standard imaging technique for the assessment of NACT response in BC. A recent meta-analysis showed that MRI had significantly higher accuracy for the detection of residual cancer with a median sensitivity of 92% but a median specificity of 60% for the detection of pCR and an NPV of 88% [24]. The NPV rate associated with MRI in pCR prediction is, however, highly variable in the literature from 44% to 96%, with FNRs ranging from 4% to 56% [24, 27]. The sensitivity and specificity of positron emission tomography-computed tomography (PET-CT) in predicting breast tumor response to NACT in a meta-analysis that pooled results of 19 studies and included 920 patients were 84% and 66%, respectively [28]. The accuracy of PET-CT for prediction of pCR showed a lot of interstudy variability for NPV, ranging from 12% to 86%, and differences in imaging performance across BC subtypes were observed [29].

Based on our findings, the use of ICG-FI on breast surgical specimen slices and/or specimen samples (blocks) could represent a complementary technique to guide and to standardize pathological evaluation of BC after NACT. The NPV and FNR of ICG-FI were 92.7% and 5.8%, respectively, and, thus, ICG-FI might predict pathologic tumoral response in a sufficiently accurate way to avoid pathological sampling of breast surgical slices that are not fluorescent. In addition, it was hypothesized that ICG-FI could allow the pathologist to focus attention on additional breast specimen slices that present with a hyperfluorescent appearance. However, this was not supported by our findings on the serial section of PE blocks that presented with hyperfluorescence without evidence of the presence of tumoral cells on correspondent pathologic slices. These serial sections guided by fluorescence were not useful for detection of residual tumoral tissue. This limitation is related to the low Sp (31.7%) and to the high FPR (68.3%) of the technique. These results can be partially explained by the inflammatory changes that occur in the breast tissue due to NACT, and/or foreign bodies (like pre-treatment tumor marking). Moreover, benign breast lesions may be, to some extent, responsible for the appearance of hyperfluorescence. An example of the limitations of our technique, but also of the difficulty of the pathological examination of breast surgical specimens after NACT, is our detection of a residual invasive tumor focus on additional serial sections performed on a hyperfluorescent sample (block) with an initial negative pathological slice. This discovery is likely to be just a fortuitous discovery of a residual focus among the ‘Swiss cheese’ pattern of tumor response, characterized by scattered microscopic residual viable tumour foci, rather than tumoral ICG-related fluorescence from the sample.

Another interesting finding in our study is the cut-off value of TBFR (1.3: obtained with Fluobeam camera) that was determined to have the best agreement between the visual (clinical) and quantitative scales of fluorescence intensity in the “ex vivo” setting. This corresponds to the value of the TBFR threshold determined (with PDE camera) in the “in vivo” FI evaluation in a previous study [12,14]. This may indicate the value of human eye threshold detection for FI, without depending on the cancer type, type of evaluation (“in vivo” or “ex vivo”), and FI camera (PDE or Fluobeam). We must take this into account in future studies of tumor cell analysis with ICG-FI.

Our study has several limitations. The first one is represented by the small number of breast tumors analyzed (with the related patient and tumor characteristics that may influence FI). In this study, we used ICG-FI to evaluate tumor changes after NACT on breast surgical specimen samples (blocks) but we did not evaluate breast tumors as a whole as was done for the assessment of NACT response in BC by imaging technique and by pathological reports. This could be a source of error for our technique of ex vivo ICG-FI for the final evaluation of the patients’ breast tumor response due to differences in response to NACT related to tumor heterogeneity. Future studies of ICG-FI evaluation of residual tumor tissue after NACT with a larger number of patients are needed to better clarify this very important point. The second limitation is represented by the standard selection of the pathological specimens in each patient which did not take into account the “extra” hyperfluorescent samples (which might have a possible impact on disease plan change management if ICG-FI residual malignancy would have been identified in addition to the standard pathological examination).

Additional limitations of our exploratory study are related to the underlying pathophysiological mechanism of ICG, the timing of ICG-FI, and the statistical analysis. The exact pathophysiological mechanism of ICG-FI in the detection of malignant breast tissue and the best timing for performing ICG-FI imaging in this setting remain uncertain. The EPR effect is assumed to be the mechanism of action here, as is hypothesized for other malignancies [15]. In our study, the median time observed between ICG injection and the resection of the operative specimen was 90 minutes, corresponding to the optimal delay for ICG-FI seen in other oncological conditions [10–14]. Finally, this ‘‘proof of concept” study is limited in terms of methodological aspects related to the use of “per block” ICG-FI analyses which evaluated breast surgical specimen sample blocks and not the breast tumors as a whole. This factor, and the limited number of patients included in this study, impeded our ability to perform a multivariate analysis. These small samples of breast tumors are not representative of the breast tumor population in terms of diversity of morphological (histologic type, tumor grade) and immunohistochemical (Ki67 index, estrogen, progesterone, HER expression) characteristics, making a functioning model of ICG-FI to differentiate malignant breast tissue and benign breast tissue very difficult to build.

In conclusion, these first observations indicate that the ex-vivo use of ICG-FI in the detection of residual tumoral tissue in patients treated for BC after neoadjuvant chemotherapy is sufficiently sensitive, but not specific enough to discriminate between benign breast tissue and malignant residual tissue. Nevertheless, the negative predictive value of ICG-FI seems sufficiently accurate to identify breast operative specimen with pCR after NACT representing an interesting tool for future appropriate pathological assessment and pathology reporting of residual breast disease in surgical specimens. Further studies on larger population are needed to confirm our results.
